# Study Protocol for an Ecological Momentary Assessment Study: TempRes “Temporal Variability of Risk and Resilience Factors for Suicidal Ideation”

**DOI:** 10.3389/fpsyt.2022.877283

**Published:** 2022-04-25

**Authors:** Mareike Ernst, Ana N. Tibubos, Thomas Kubiak, Rory C. O'Connor, Manfred E. Beutel

**Affiliations:** ^1^Department of Psychosomatic Medicine and Psychotherapy, University Medical Center of the Johannes Gutenberg-University Mainz, Mainz, Germany; ^2^Diagnostics in Healthcare & E-Health, University of Trier, Trier, Germany; ^3^Health Psychology, Institute of Psychology, Johannes Gutenberg-University Mainz, Mainz, Germany; ^4^Suicidal Behaviour Research Laboratory, Institute of Health and Wellbeing, University of Glasgow, Glasgow, United Kingdom

**Keywords:** Ecological Momentary Assessment, loneliness, personality functioning, risk factors, suicide, suicidal ideation

## Abstract

Suicide prevention is an important public mental health issue that can be significantly brought forward by recent advances in psychological research methods and assessment. The project “TempRes” aims to harness the power of Ecological Momentary Assessment (EMA) to investigate the transdiagnostic risk and resilience factors associated with suicidal ideation drawn from the most recent research in suicide prevention and personality assessment. Participants will comprise the general population (planned: *N* = 100) and a risk group (patients currently in psychosomatic or psychiatric treatment) (planned: *N* = 50). After a comprehensive baseline assessment, they will complete up to ten short assessments per day over the course of 10 days at roughly equidistant intervals. In detail, the project examines the interplay of biography (previous suicidal behavior, experiences of childhood maltreatment), individual differences (level of personality functioning), and time-varying factors (entrapment, loneliness, mood) with respect to the emergence and fluctuation of suicidal ideation. There are two main research foci: First, the project will provide an operationalization and empirical verification of a core assumption of the *integrated motivational-volitional model of suicide* (IMV model). It will test whether the interaction of the time-varying predictors entrapment with loneliness (as a motivational moderator) explains reports of suicidal ideation over time. Second, it will be the first to examine *personality functioning* (a transdiagnostic, psychodynamically grounded conceptualization of vulnerability to psychological crises over the life span) as a time-invariant predictor of suicidal ideation assessed within an intensive longitudinal study design. The main analyses will be built on linear mixed models. The overarching aim of the project is to gain a better understanding of the psychological dynamics underlying suicidal ideation in different populations by bringing together concepts from different theoretical traditions. This will inform prevention efforts geared toward the general public as well as intervention in clinical populations.

## Introduction

Suicide is a global mental health issue that affects all strata of the population. It is estimated that over 700.000 people die by suicide every year (1). Despite decades of research and efforts to improve mental health care, persistent, or even rising numbers in some countries [e.g., (2, 3)] underline that suicidal ideation and behavior still pose great challenges. Furthermore, syntheses of empirical investigations of risk factors have not yet provided for the design of individual risk algorithms with satisfying accuracy (4). This is in part because of the relatively low case numbers of suicide outcomes in the general population, but also because of their complex, biopsychosocial etiology and challenges associated with their assessment. In the following, we describe how the most recent empirical evidence suggests that relevant progress in suicide research can be achieved through study designs that implement the following insights: first, multiple risk factors and/or protective factors work together, second, suicidal ideation and behavior are distinct entities, and third, suicidal ideation can fluctuate substantially. Going from there, we describe how the project TempRes (“Temporal variability of risk and resilience factors for suicidal ideation”) aims to make a contribution to the field by also integrating social-cognitive research traditions and psychodynamic concepts.

**Figure 1:**
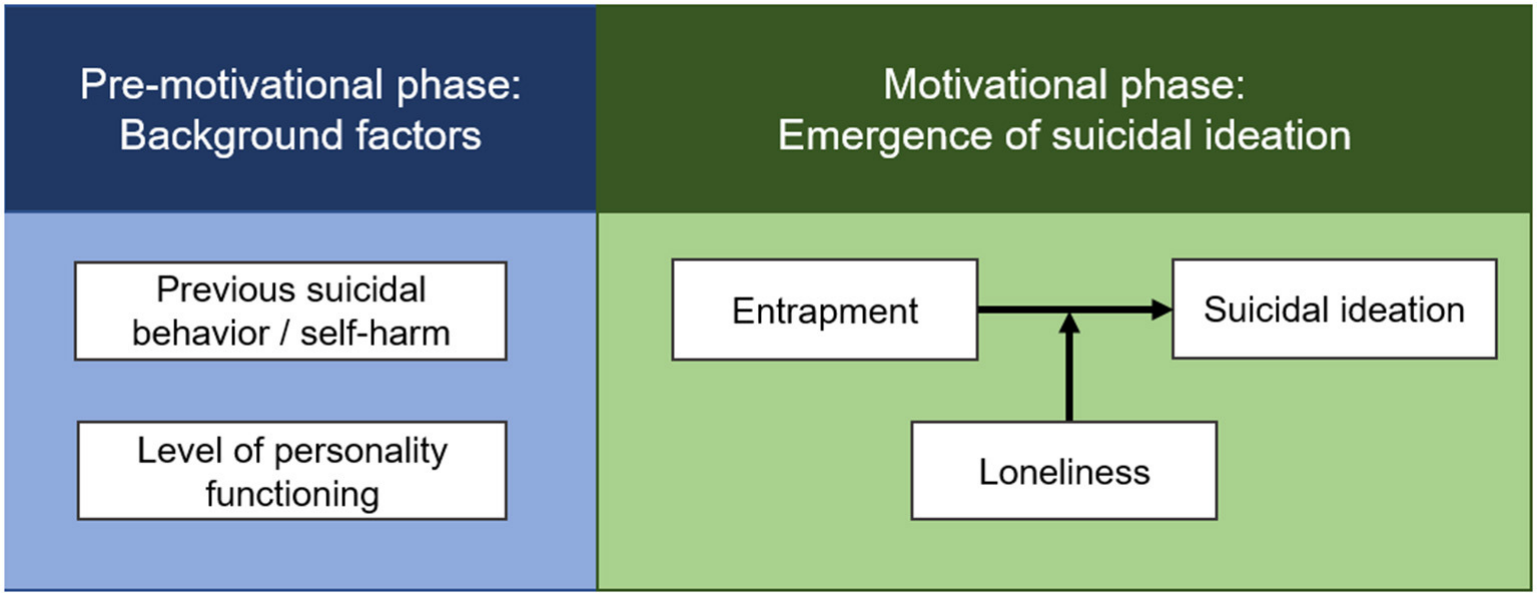
Simplified depiction of the variables and associations that are the focus of the primary research goal. A reduced, specified version of the integrated motivational-volitional model of suicide [IMV model; O'Connor and Kirtley (12)] will be tested in the context of the project. The IMV model differentiates three phases: First, the pre-motivational phase describes the broader context of suicidal crises over the life span. Second, in the motivational phase, suicidal ideation is conceptualized as the result of the dynamic interplay of distressing emotional states and moderating factors that determine an individual's ability to cope with them. Lastly, the transition to suicidal behavior—the volitional phase—is shaped by further, specific risk factors (the volitional phase is not depicted here as the project focuses on suicidal ideation). All motivational phase variables will be assessed using Ecological Momentary Assessment.

First, many epidemiologically-oriented studies have tested associations of suicide outcomes with other variables (4–6). However, a large proportion of risk indicators established this way are imprecise, for example, because they apply to large groups of the population [such as male gender). In addition, studies have reported distal risk factors (such as child maltreatment (7)] that might not directly exacerbate the risk of suicidal crises as their effects are altered by further mediating and moderating variables. Although necessarily reductionist study designs cannot possibly mirror the complexity underlying the emergence of suicidal crises in daily life, new insights could be gained by studying different risk factors in combination (8, 9). Respective statistical operationalizations could include stratified analyses (e.g., 10) which can give insight into group-specific risk constellations. Another option is the modeling of two (or more) variables' interplay using interaction terms [e.g., (10)]. Corresponding models could not just include relatively stable individual differences, but also time-varying environmental variables or mental and emotional states. This seems particularly appropriate against the background that psychological science has long conceived of distress or wellbeing, respectively, as the result of an interplay of the person and the situation: In Lewin's famous formula [B ~ (P;E)], behavior (B; extended to cognitions and emotions) is a function of the person (P) and their environment (E) (11). Previous research has highlighted important factors within each of these domains. A particularly comprehensive and differentiated synopsis is presented by the integrated motivational-volitional (IMV) model of suicide (12). The IMV model differentiates three phases [see Figure 1 in O'Connor and Kirtley (12) for a visual depiction]: The pre-motivational phase describes the broader context of biological, psychological, and social vulnerability and resilience factors shaping the risk of suicidal crises over the life span, analogous to classical diathesis-stress-models. It is followed by the motivational phase. The conceptualization of this second phase emphasizes that the emergence of suicidal ideation is a highly dynamic process involving the interplay of various factors, most of which are psychological in nature: If an individual experiences defeat or humiliation, their risk of suicidal ideation further hinges on their ability to cope with these feelings in an adaptive way (according to individual differences summarized under threat-to-self moderators). If they do not succeed in resolving this distressing subjective state, they could feel entrapped, meaning they see no way out. At this point, motivational moderators, such as social support, influence whether the individual can think of alternative ways to end their emotional pain (rather than ending their life). Lastly, the model includes a volitional phase in which self-destructive thoughts are acted upon (i.e., the person engages in suicidal behavior). Factors that govern the transition from the motivational to the volitional phase are called volitional moderators. As an example, they comprise access to means, i.e., suicide attempts will be facilitated if the person at risk has a firearm in their home.

Second, the distinction between suicidal ideation and behavior inherent in the IMV model must be emphasized. It is also a central part of two other current theories of suicidal ideation and behavior within the influential ideation-to-action framework (13), the Interpersonal Theory of Suicide (IPTS) (14) and the Three-Step Theory of Suicide (3ST) (15). This distinction reflects the state of the art of suicide research which has shown that risk factors for suicidal ideation and behavior are distinct (16, 17). Variables that govern the transition from the former to the latter are called volitional moderators within the IMV model and comprise e.g., access to means such as firearms (17). While suicidal behavior comprises non-lethal as well as lethal suicide attempts, suicidal ideation can manifest as passive (i.e., death wishes) or as active (i.e., the desire to engage in suicidal behavior). An instructive summary and guide of how different suicide outcomes should be denominated is provided by Silverman, Berman (18). To yield meaningful results that others can build on, it is important for research endeavors to clearly define and correctly assess the respective suicide outcome of interest instead of conflating different manifestations (e.g., by summarizing them under the umbrella term “suicidality,” which muddles the waters and hampers progress) (9).

Third, as noted above, previous research on suicidal ideation and behavior has provided a lot of information describing *who* is at risk (e.g., men, individuals who have experienced childhood abuse). By contrast, an important frontier concerns the question of *when* somebody is at risk (i.e., within-person variation). Within the context of suicide research, this is an especially meaningful question as suicidal crises have been shown to be transient states (19, 20). Hence, fluctuating suicidal ideation is not captured well by questionnaire assessments that instruct individuals to report mental distress over the course of the last weeks (either as an average or as days in a week on which they suffered from specific symptoms). In such cases, responses are also influenced by the emotional state in the here and now (21) and recall biases (22). For instance, reports of suicidal ideation which were made during the week did not always match responses in a questionnaire administered at the end of the week (using established instruments such as the Beck Scale for Suicide Ideation) (23). What is more, risk factors for suicidal ideation also vary over time, e.g., defeat and entrapment, central aspects of the motivational phase of the IMV model (24). Thus, a valid assessment of suicidal ideation and associated risk or protective factors needs to be change-sensitive. This applies to the questions which are asked (e.g., “How do you feel now?,” instead of “How did you generally feel over the course of the last month?”) and to the sampling method (e.g., multiple times a day instead of once every few weeks). Research designs that enable the study of psychological processes as they unfold in daily life are called ambulatory assessment (AA), Ecological Momentary Assessment (EMA), or daily life methods (25). Considerable technological advances in recent years have made them easier to administer, for example by using apps on participants' mobile phones. Consequently, there is now a large body of research using these methods in healthy and clinical populations, including individuals at risk for suicidal ideation and behavior (20, 26). The proportions of participants reporting suicidal ideation varied widely between different studies with intensive longitudinal designs, ranging between a fifth of participants and the entirety of participants (27). However, sample characteristics (such as whether participants were drawn from the community or a clinical sample) were more closely associated with studies' detection of suicidal ideation than the number of daily assessments or other design characteristics. Within the present study, we use the term EMA as it does not include any passive monitoring (e.g., of biomedical parameters), but the real-world and real-time aspects of the assessment are deemed the most important following Silvia and Cotter (25).

Lastly, there are seminal psychodynamic contributions to the understanding of both “normal” development and psychopathology, including suicidal crises [e.g., (28–30)]. In spite of attempts at building bridges between disciplines [e.g., (31, 32)], so far psychodynamic approaches have not been brought together with the major developments in the empirical study of suicidal ideation and behavior described above. However, recent advances in the operationalization of central psychodynamic concepts facilitate the integration of these perspectives. In particular, the project will investigate the role of the level of personality functioning. As a dimensional ascertainment of temporally stable, basic abilities or impairments, respectively, it has long been an important component of psychodynamic diagnosis and treatment planning. Since the reconceptualization of personality pathology in the upcoming eleventh revision of the International Classification of Diseases (ICD-11) and the DSM-5 Alternative Model for the Assessment of Personality Disorders (33), it has received increased attention. Besides informing about broad, health-related personality characteristics, the assessment of personality functioning could yield new insights into the between-and within-person factors underlying suicidal ideation. First empirical studies in patient samples (34, 35) that highlighted its transdiagnostic relevance for different suicide outcomes support this notion.

Building on previous research and following the considerations outlined above, the project TempRes uses an intensive longitudinal study design and focuses on suicidal ideation. Study assessments include the pre-motivational and the motivational phase of the IMV model: The more extensive assessment at study intake captures constructs that are assumed to be relevant time-invariant risk or protective factors, such as the level of personality functioning. In the following EMA module, assessments focus on psychological state variables (e.g., entrapment, loneliness) and the context (e.g., where participants are at the moment, whether someone is with them).

The primary research goal is the operationalization and empirical verification of a core assumption of the IMV model over time (Figure 1 depicts the main constructs of interest).

For this research goal, we will test the following hypotheses:

Hypothesis 1: According to premise 6 of the IMV model (detailed in 13), individuals who have intentionally self-harmed in the past report higher levels of the motivational phase variables (entrapment, loneliness, and suicidal ideation) than those who have not done so.
Hypothesis 2: Motivational phase variables (entrapment, loneliness, and suicidal ideation) vary over time.
Hypothesis 3: Entrapment is positively associated with suicidal ideation:
Hypothesis 3.1 Feelings of entrapment are positively associated with suicidal ideation.
Hypothesis 3.2 The relationship between entrapment and suicidal ideation is moderated by loneliness, i.e., it is particularly strong if people also feel lonely.

The secondary research goal concerns the exploration of the role of personality functioning. Since this part of the project cannot directly relate to previous studies with similar methodology, the hypotheses are less concrete: First, we will explore associations of personality functioning with established risk and protective factors from the pre-motivational phase (e.g., we expect impaired personality functioning to be associated with childhood abuse and neglect and with previous self-harm). Second, the level of personality functioning will be tested as a background factor, in the sense that greater impairments are assumed to be associated with higher levels of the motivational phase variables. However, it could also play a role as a moderator within the motivational phase: motivational moderators are defined as “factors that, when present and protective, allow the trapped individual to see alternatives, a more positive future and less pain” (12). Respective constructs included implicit and explicit attitudes as well as negative self-appraisals, both of which conceptually overlap with measures of personality functioning such as the OPD-Structure Questionnaire (OPD-SQS) (36). In addition, studies have suggested an intermediate role of personality functioning, for instance, it linked childhood abuse and neglect with mental distress later in life in a cross-sectional population study (37) and with inhibition difficulties in an experimental go/no-go task (38) in patients with depressive and personality disorders and healthy control participants.

## Methods and Analysis

This is a prospective, observational study with an intensive longitudinal design. Participants are recruited from the general population and patients treated at the University Medical Center Mainz (Department of Psychosomatic Medicine and Psychotherapy, Department of Psychiatry and Psychotherapy).

### Selection of Participants

With the combination of a population sample and a patient sample, we aim to achieve variation regarding the variables of interest: On the one hand, rates of suicidal ideation (assessed using EMA) and behavior (assessed retrospectively) in the general population are relatively low (e.g., 9). In a recent survey of a representative German community sample, we found rates below 10% (using the PHQ-9 item) (39) whereas an analysis of the same item based on unpublished routine data of 724 patients treated at the Department of Psychosomatic Medicine and Psychotherapy of the University Medical Center Mainz over the course of 4 years showed rates of 41%. They are considerably higher in clinical populations. On the other hand, individuals in psychiatric and psychosomatic-psychotherapeutic (inpatient) treatment show comparatively greater impairments in personality functioning (40). As we intend to study associations of personality functioning with suicidal ideation and established risk and protective factors (including more complex associations proposed by previous research, such as moderating or mediating effects), it will be beneficial to recruit a substantial number of mentally healthy individuals with higher levels of personality functioning as well.

Inclusion or exclusion criteria, respectively, are the same for the general population and the patient sample. Given that the study focuses on transdiagnostic risk and resilience factors, participation will be open to a broad range of individuals. However, participants need to be able to understand and complete the assessments as intended. This is why individuals with current substance use disorders and psychotic symptoms are not considered eligible. Further exclusion criteria are insufficient knowledge of the German language and the absence of a smartphone for personal use. Lastly, the study focuses on the motivational phase in which suicidal ideation arises. It does not include the study of (risk and protective factors associated with) suicidal behavior/self-harm. As the technical realization of the study neither allows for the personal identification of single participants by the research team nor for the processing of data in a way that would allow for real-time-monitoring and intervention. Therefore, we decided that for safety reasons, acutely suicidal individuals cannot take part.

### Recruitment and Consent Procedure

The recruitment of participants of the *population sample* will predominantly use online channels. A short description of the study and contact information will be posted online (e.g., on the website of the Department of Psychosomatic Medicine and Psychotherapy of the University Medical Center Mainz and the Study Center for Clinical Trials in Mental Disorders of the University Medical Center Mainz) and circulated via social media. In order to recruit *patients*, the same information will be included in a leaflet that will be distributed in waiting areas and/or handed directly to patients (in treatment at the Department of Psychosomatic Medicine and Psychotherapy or the Department of Psychiatry and Psychotherapy of the University Medical Center Mainz) by clinical and research staff. Additionally, posters will be distributed on the clinic premises. We will actively seek to oversample male patients to achieve a gender-balanced sample, as patients treated in mental health care, e.g., at the Department of Psychosomatic Medicine and Psychotherapy are predominantly female.

After a short eligibility check, individuals will be informed about the study in detail (patient sample: in person by the Principal Investigator (PI) or a research assistant; population sample: online in writing or, if desired, also by phone). All eligible individuals will be informed that data will be gathered and analyzed anonymously and that they can withdraw from the study at any time without explanation without any negative consequences. They will have the opportunity to ask questions. Only then will they be asked to fill out an informed consent form (patient sample: on paper, a copy will remain with them; population sample: online).

### Ethics and Safety Aspects

The study was designed in line with expert recommendations for intensive longitudinal studies (including in at-risk populations) (41, 42). It follows guidelines for good clinical practice and fulfills the ethical principles of the declaration of Helsinki. Data is collected in a parsimonious, anonymous way combining the Samply Research app (43) with the SoSci Survey platform (44). Participants are never asked to enter identifying information such as their names. All procedures are in line with the General Data Protection Regulation (GDPR). Participant information and consent forms were created in line with the recommendations of the German Psychological Society (DGPs). The study contents and procedure were approved by the ethics committee of the Department of Psychology of the Johannes Gutenberg-University Mainz (number 2021-JGU-psychEK-019).

Before participants are enrolled, a feasibility study will be conducted (*N* = 5–10) to test the technical implementation of data collection and management. Only afterward will participant recruitment start, first for the population sample.

The study has been registered in the research registry of the University Medical Center Mainz (Forschungsregister FoR.UM) which is managed by the interdisciplinary center for clinical trials (Interdisziplinäres Zentrum Klinische Studien, IZKS) (number 21-00292). Registration includes information about the status of the project and the responsible PI and research staff. It allows the site management organization to track the numbers of screened, included, and excluded participants.

The study is observational and does not include any interventions. However, it deals with distressing psychological states. This is why the first page of the online questionnaire administered in the population sample contains information about emergency contacts (e.g., the German telephone counseling service Telefonseelsorge). Patients will be in the clinic setting for the duration of the study, where a safety protocol is in place (standard operating procedures for keeping acutely suicidal patients safe, developed by trained staff that includes clinicians and researchers with decades-long experience in working with individuals with severe mental disorders).

Along these lines, we find it important to rebut persistent “suicide myths” that asking about suicidal ideation would amplify a suicidal crisis (i.e., that it is iatrogenic). This is not the case: Previous research has found that confrontation with respective stimuli did not contribute to higher levels of suicidal ideation. Disclosure of suicidal ideation was instead associated with relief (45). Repeated assessment of suicidal ideation in the context of AA studies did not increase suicidal ideation either (46). However, it has been noted that digital monitoring studies of mental health outcomes pose particular ethical issues, such as whether researchers should intervene, and if so, how, if participants report suicidal ideation or self-harm (impulses) (42, 47). There are no established best practices yet, but according to a recent review, sixty percent of previous studies used automated pop-up notifications while forty percent of studies did not monitor participants' responses (47). While experts have recommended daily monitoring in at-risk populations (41), the technical implementation of data collection and lack of resources to screen and adequately react to incoming data in the present study did not allow for real-time monitoring either. Therefore, we decided to exclude acutely suicidal individuals from participation due to safety concerns.

### Study Assessments

**Figure 2:**
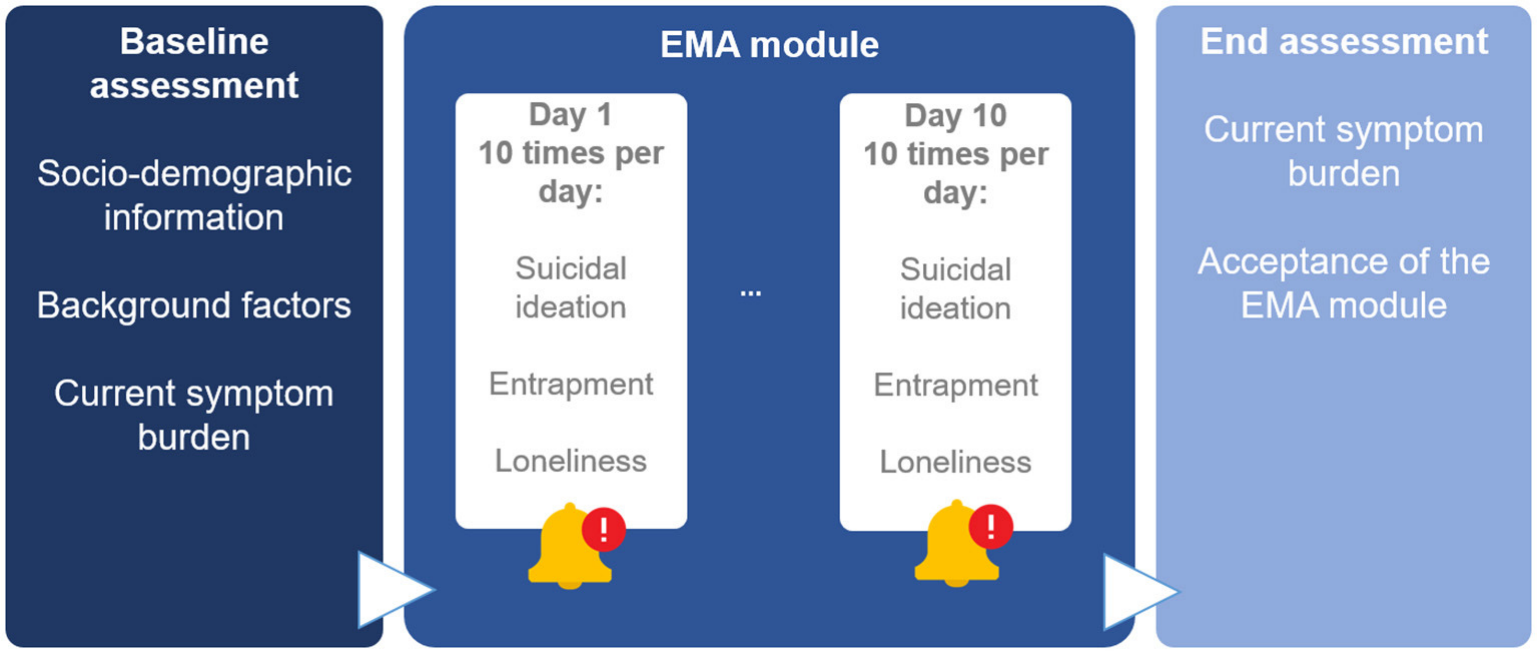
Depiction of assessments within the project TempRes. The signal-contingent Ecological Momentary Assessment (EMA) module contains ten assessments per day over a course of 10 days, resulting in up to 100 single assessments per person. Displayed are the main constructs of interest in the context of the primary research question. Beyond these, the EMA module also includes assessments of mood and context variables.

Study assessments comprise three major modules (shown in Figure 2): A comprehensive baseline assessment at study intake (before the first day of EMA), an EMA module (spanning 10 days, with ten assessments per day), and an end-of-study assessment (after the last day of EMA).

Before the baseline assessment, participants generate their participant code which is used to link all modules. After the baseline assessment, they receive a short instruction concerning the app Samply Research through which the EMA module and the end-of-study assessment are controlled. After signing up and entering the participant code once, participants no longer have to open the app independently as assessments are prompted by notifications.

#### Baseline Assessment

This assessment captures socio-demographic information (such as gender, age, level of education, living situation, and employment), whether participants were recruited from the general population or in the context of their treatment at one of the two participating departments of the University Medical Center Mainz, and a concise survey of self-reported technical/digital competencies. It also includes established, psychometrically sound inventories assessing background factors as well as currently relevant stressors and domains of distress:

a. Current stressors, symptoms, and emotional states: PHQ Stress, PHQ-9, PHQ-15, GAD-7, which are all included in the Patient Health Questionnaire (48, 49), Mini Social Phobia Inventory (Mini-Spin) (50), Beck Scale for Suicide Ideation (51), Beck Hopelessness Scale BHS (52), Subjective emptiness scale (SES) (53), Short Defeat and Entrapment Scale (54, 55), Brief Reasons for Living Inventory (BRFL) (56)
b. Social factors: Brief Social Support Scale BS-6 (57), UCLA 3-Item Loneliness Scale (58, 59), Experiences in Close Relationships (ECR-RD-8) (60), Interpersonal Needs Questionnaire (INQ) (61)
c. Individual differences and personality characteristics: Scale Optimism-Pessimism-2 (SOP2) (62), Emotional Regulation Questionnaire (ERQ) (63), Theoretical Depressive Experiences Questionnaire-12 (TDEQ-12) (64), Big Five Inventory (BFI-10) (65), OPD-Structure Questionnaire (OPD-SQS) (36), Theoretical Depressive Experiences Questionnaire TDEQ-12 (64)
d. Experiences while growing up: Recalled parental rearing behavior (66), Childhood Trauma Questionnaire CTQ (67)

In addition to these inventories, the assessment includes questions about pandemic-related stressors (e.g., current quarantine). Although the study does not aim to examine mental health in relation to the COVID-19-pandemic, this context cannot be ignored at the present time.

#### EMA Module

In Sample Research, notifications are planned for all 100 scheduled assessment of the EMA module and for the end assessment. The former are sent via push messages at preset times (pseudo-randomized intervals between 8 a.m. and 10 p.m., with a minimum interval of 45 min) to each participant. Each push message contains the same link to the EMA survey on SoSci Survey. Respondents need only click on the link and answer the questionnaire in their browser.

Each assessment includes fewer than 25 items in total. All items except the context variables will use the same response format (1 = “not at all” to 7 = “very”). Context items will assess where participants are currently (e.g., at home, at work) and whether they are alone or have company. Suicidal ideation will be measured using three items adapted from the Beck Scale for Suicide Ideation (51) (“I have a wish to die,” “I have a wish to live,” “I have a wish to kill myself”). As suicidal ideation is not a uniform construct (see Introduction), this kind of assessment allows for the differentiation of a wish to die (i.e., passive death wishes), wish to live, and wish to end one's life (i.e., active suicidal ideation). It will be placed in the middle of the administered items to reduce participants' reactivity to it. The same applies to defeat, entrapment [assessed with two items each, drawn from the German Defeat Scale (DS-d) (68) and the German Entrapment Scale (ES) (69)] and loneliness [assessed with two items, adapted from Kleiman, Turner (19)]. Additional items include positive and negative affect, which will be measured using items drawn from the German PANAS-X (70, 71). Lastly, two items drawn from the Beck Hopelessness Scale (52) will assess hope/hopelessness.

Items were chosen based on their psychometric properties (e.g., those with the highest factor loadings of their respective scales in the questionnaire long form). In cases where items were drawn from longer scales, we checked whether their wording needed to be adapted to the “here and now.” However, this was not necessary as the original questionnaires communicated the time period of interest as part of the instruction rather than as part of the items themselves. The sequencing of items does not change throughout the study.

#### End Assessment

The end assessment repeats the most important symptom measures from the start assessment. It includes questions about special events in the last days that the researchers should be aware of when interpreting the data (answered via free text). It also assesses the acceptance of the EMA module (regarding both the contents and the method) and the perceived intrusiveness of the EMA protocol.

### Data Structure and Analyses

Analyses will be conducted using R (72) and mainly using the packages esmpack (73), lme4 (74), and lmerTest (75).

The special survey methodology implicates a longitudinal and nested data structure in which the measurement points (Level 1) are nested within days (Level 2) which are in turn nested in participants (Level 3). This is why the main analyses will build on linear mixed models, a technique in which separate regression functions are estimated for each cluster. Such models expand the standard regression model by random effects that allow for the investigation of person-level differences in model coefficients [see e.g., (76, 77)]. Models will include random intercepts and random slopes (i.e., the deviations of participants' values from the respective group mean) and account for autocorrelation. Moderation effects will be probed by creating the product of the mean-centered variables of interest (entrapment and loneliness). In the case of statistically significant interaction effects, the steepness of the regression line on different values of the moderator (i.e., entrapment) will be visualized (e.g., for the sample mean and the sample mean +/– one standard deviation).

The primary outcome is suicidal ideation. Its operationalization via three items allows for tests of construct validity and reliability [e.g., as nested α (78)]. Secondary outcomes are entrapment and loneliness.

#### Expectations Regarding Compliance

Review articles of previous EMA studies found compliance rates of 79% across samples (79) and 77% in studies focusing on self-harm and suicide (26). However, as suicidal ideation, the main construct of interest, has been shown to fluctuate substantially (19, 80), we chose a sampling design with more assessments per day and a longer assessment period than most of the summarized research (e.g., respective mean values were six assessments per day over the course of 1 week (79). Therefore, we expect compliance rates in the present study to be lower, i.e., around 60 of 100 assessments. Studies achieved higher compliance rates when using incentives (79). In this study, we do not communicate a certain target compliance rate to participants (e.g., the completion of >50% of assessments) as we do not want to motivate them to answer prompts without regard to the questions/answering truthfully. However, they are informed that all participants who stay with the study for 10 days are—at the very end—given the opportunity to enter a raffle (of 15€-shopping vouchers for a supermarket). After data collection is completed, individual participants' data might be excluded from the analyses if the free text answer in the end assessment contains information about events that complicate the interpretability of their responses in a substantial way (e.g., technical dysfunction of their mobile phone). Such cases will be made transparent in the study reports. It is also common practice to include only individuals with a certain percentage of completed assessments in the analyses. However, all thresholds (e.g., 15%) applied in this context are somewhat arbitrary. Therefore, we will follow recommendations to only use them in combination with sensitivity analyses using the whole sample, in order to check whether the resulting findings are robust (81). Predictors of signal compliance will also be investigated (e.g., age, gender, symptom burden, and technical/digital competencies).

#### Power and Sample Size Considerations

Considerations of power and sample size in intensive longitudinal studies must include both within- and between-person sources of variation. These sources of variation are influenced differently by the number of measurement points and the number of participants included in the study, complicating calculations (76). This might be one of the reasons why reports of intensive longitudinal studies seldom include power calculations or sample size justifications (82). We conducted general power and sample size calculations for a longitudinal study design (83) targeting outcome variation within participants, in which


$$\begin{array}{l} Power=\frac{n\;x\;m}{1+\left(m-1\right)\;x\;ICC} \end{array}$$

with n indicating number of participants and m indicating measurement points per participant. The intraclass correlation (ICC) states how much variance of a variable is due to temporally stable factors, i.e., between-person variability as opposed to within-person variability (76). This formula can be transformed the following way:


$$\begin{array}{l} n=\frac{Power\;x\;\left(1+m-1\right)\;x\;ICC}{m}\; \end{array}$$

In previous studies, the ICC of the motivational phase variables of interest ranged between 0.54 [for entrapment (24)] and 0.67 [for suicidal ideation (19)], so we chose the upper bound for our calculation. With a desired power level of 80%, 60 expected assessments per person (see above), and an ICC of 0.67, we would need 54 participants overall.

However, given the fact that the estimated compliance rate is not guaranteed and we aim to test several hypotheses, e.g., regarding group differences concerning pre-motivational phase variables, we plan to recruit more participants than the calculated minimum sample size. Therefore, we plan to assess about 50 patients and 100 participants from the general population (as recruitment of the latter is less costly in terms of research staff's time). Empirical investigations of the single hypotheses, including cross-level interactions, will include more specific, simulation-based power analyses making use of resources such as the recently developed app PowerAnalysisIL (84).

## Discussion of Strengths and Limitations

In summary, the main objectives of the TempRes study concern the validation of a part of the established, influential IMV model and the integration of powerful, empirical methods allowing for the modeling and differentiation of between- and within-person effects with recent developments in the operationalization of the level of personality functioning. Building on current research advances, the project aims to contribute to a better understanding of suicidal ideation in both at-risk groups and the community to inform prevention and intervention efforts. A recent systematic review (27) showed that assessing both passive and active suicidal ideation—as this study will do—increased detection rates of suicidal ideation. Further, the EMA module includes both theoretically-based, specific constructs [such as entrapment, e.g., (85)] and broader categories of subjective experience [such as negative affect, e.g., (20)] that were associated with suicidal ideation in previous research. The present study is also implemented in such a way that both participant data privacy and open science principles, such as the provision of anonymized study data, have a high priority.

However, there are several limitations. As mentioned, low proportions of individuals reporting suicidal ideation, especially in non-clinical populations, present a challenge to empirical research on associated risk and protective factors. The present study tries to address it by combining different samples. However, if responses on the respective items yield a highly skewed distribution, it might be necessary to adapt the analysis strategy (either by calculating logistic mixed-effects models or by summarizing instances of any level of suicidal ideation). Further, anonymity in responses and the absence of incentives/guaranteed reimbursement might hamper compliance and implicate comparatively low participation rates. As the EMA module only assesses suicidal ideation, the study cannot give insight into time-varying risk and protective factors underlying suicidal behavior. The exclusion of acutely suicidal individuals (which we deemed necessary for safety reasons) represents a further limitation as it means that the resulting findings might not apply to those individuals with the highest risk. In future research with the appropriate resources, they should be included, and their responses should be closely monitored (41). Indications of acute risk should also receive some sort of reaction. However, as it is unclear whether pop-up notifications are read and perceived as helpful in case of acute risk (47), ideally, trained staff should be available to reach out to participants, e.g., by phone.

Lastly, it will not be feasible to recruit a representative sample of the population or of the patients treated at the participating departments. There might be specific self-selection effects (including motivational aspects and expectations of the study) which we can neither assess nor statistically control for. However, we intend to compare study participants' characteristics (e.g., gender distribution, age, level of education, symptom burden) with both routine data collected on all consecutive patients treated at the clinics during the trial period and representative survey data of the general population.

## Author Contributions

ME and MB: conceptualization. ME: writing—original draft and preparation. AT, TK, RO'C, and MB: writing—review and editing. All authors contributed to the article and approved the submitted version.

## Funding

Funding was provided through a grant of the Mainz Research Center for Mental Disorders' Early Career Program (MZPG Early Connect) awarded to ME.

## Conflict of Interest

The authors declare that the research was conducted in the absence of any commercial or financial relationships that could be construed as a potential conflict of interest.

## Publisher's Note

All claims expressed in this article are solely those of the authors and do not necessarily represent those of their affiliated organizations, or those of the publisher, the editors and the reviewers. Any product that may be evaluated in this article, or claim that may be made by its manufacturer, is not guaranteed or endorsed by the publisher.
